# The Measurement of the Neutron Yield of the ^7^Li(p,n)^7^Be Reaction in Lithium Targets

**DOI:** 10.3390/biology10090824

**Published:** 2021-08-24

**Authors:** Marina Bikchurina, Timofey Bykov, Dmitrii Kasatov, Iaroslav Kolesnikov, Aleksandr Makarov, Ivan Shchudlo, Evgeniia Sokolova, Sergey Taskaev

**Affiliations:** 1 Budker Institute of Nuclear Physics, 11 Lavrentiev Ave., 630090 Novosibirsk, Russia; knkstdor@gmail.com (M.B.); timaisabrony@gmail.com (T.B.); kasatovd@gmail.com (D.K.); katyono@mail.ru (I.K.); alexxmak314@gmail.com (A.M.); cshudlo.i.m@gmail.com (I.S.); evg.sokol.ol@gmail.com (E.S.); 2 Department of Physics, Novosibirsk State University, 2 Pirogov Str., 630090 Novosibirsk, Russia

**Keywords:** boron neutron capture therapy, neutron source, charged particle accelerator, lithium target, γ-radiation spectrometer, backscattering protons

## Abstract

**Simple Summary:**

A compact neutron source has been proposed and created at the Budker Institute of Nuclear Physics in Novosibirsk, Russia. The source comprises an original design tandem accelerator, solid lithium target, and a neutron beam shaping assembly. The neutron source is capable of producing the high neutron flux for boron neutron capture therapy (BNCT). Currently, the BNCT technique has entered into clinical practice in the world: two clinics began treating patients, and four more BNCT clinics are ready to start operating. The neutron source proposed at the Budker Institute served as a prototype for a facility created for a clinic in Xiamen (China). It is planned to equip the National Medical Research Center of Oncology (Moscow, Russia) and National Oncological Hadron Therapy Center (Pavia, Italy) with the same neutron sources. Due to the impending use of an accelerator neutron source for treating patients, the validation of the neutron yield of the ^7^Li(p,n)^7^Be reaction in lithium metal targets is required. The theoretical neutron yield has not been evaluated experimentally so far.

**Abstract:**

A compact accelerator-based neutron source has been proposed and created at the Budker Institute of Nuclear Physics in Novosibirsk, Russia. An original design tandem accelerator is used to provide a proton beam. The neutron flux is generated as a result of the ^7^Li(p,n)^7^Be threshold reaction using the solid lithium target. A beam shaping assembly is applied to convert this flux into a beam of epithermal neutrons with characteristics suitable for BNCT. The BNCT technique is being tested in in vitro and in vivo studies, and dosimetry methods are being developed. Currently, the BNCT technique has entered into clinical practice in the world: after successful clinical trials, two clinics in Japan began treating patients, and four more BNCT clinics are ready to start operating. The neutron source proposed at the Budker Institute of Nuclear Physics served as a prototype for a facility created for a clinic in Xiamen (China). It is planned to equip the National Medical Research Center of Oncology (Moscow, Russia) and National Oncological Hadron Therapy Center (Pavia, Italy) with the same neutron sources. Due to the impending use of an accelerator neutron source for treating patients, the validation of the neutron yield of the ^7^Li(p,n)^7^Be reaction in lithium metal targets is required. The theoretical neutron yield has not been evaluated experimentally so far.

## 1. Introduction

Boron neutron capture therapy (BNCT) [1]—selective destruction of tumor cells by accumulating boron-10 stable isotopes in them followed by neutron irradiation—is considered to be one of the promising methods to treat malignant tumors. As a result of neutron absorption by boron, a nuclear reaction ^10^B(n,α)^7^Li occurs with high energy release in the cell, which leads to its destruction. In 2020, the BNCT technique, proposed in 1936, entered into clinical practice: two Japanese clinics began treating patients after successful clinical trials [2,3]. At present, four more BNCT clinics have been built in the world, including one in China, which is equipped with an accelerator-based neutron source developed at the Budker Institute of Nuclear Physics and commercialized by TAE Life Sciences (Santa Monica, CA, USA). It is planned to equip the National Medical Research Center of Oncology (Moscow, Russia) and National Oncological Hadron Therapy Center (Pavia, Italy) with the same neutron sources.

Due to the impending use of an accelerator neutron source for treating patients, the validation of the neutron yield of the ^7^Li(p,n)^7^Be reaction in lithium metal targets is required. The theoretical neutron yield has not been evaluated experimentally so far.

## 2. Materials and Methods

**Figure 1:**
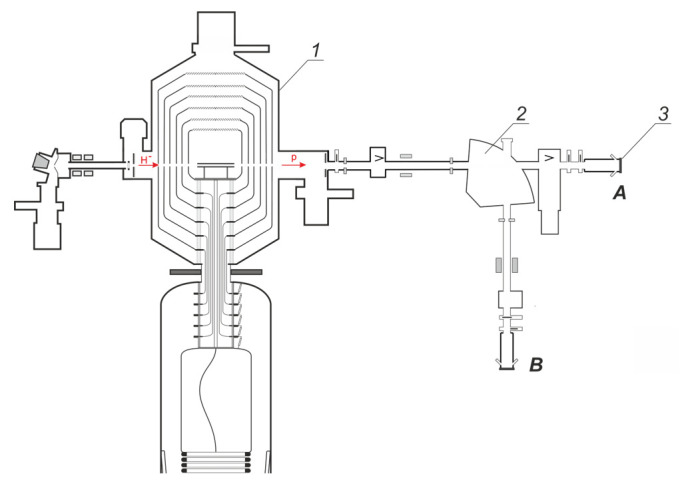
The layout of the experimental facility: *1*—vacuum insulated tandem accelerator, *2*—bending magnet, *3*—lithium target. *A*, *B*—lithium target placement positions.

Experimental studies were carried out on an accelerator-based neutron source proposed and created at the Budker Institute of Nuclear Physics, Novosibirsk, Russia [4]. The layout of the facility is shown in Figure 1. The lithium target can be placed either in the horizontal path of the proton beam transport (position A) or in the vertical (position B) with the bending magnet switched on.

### 2.1. Target Assembly and Preparation

The lithium target is a copper disk with a diameter of 144 mm and a thickness of 8 mm. On one side of the disc, in the center of a circle with a diameter of 82 mm, a visually uniform lithium layer with an average thickness of 60 to 100 μm is deposited (this value is less than the total proton path length in metallic lithium but more than the path length up to the ^7^Li(p,n)^7^Be reaction threshold). On the reverse side of the copper disk, inside a 122 mm diameter, spiral channels are made for water cooling. This side of the copper disc is pressed with a 16.5 mm thick aluminum disc. The lithium target is integrated into the target unit equipped with a gate and windows for observing the target surface.

**Figure 2:**
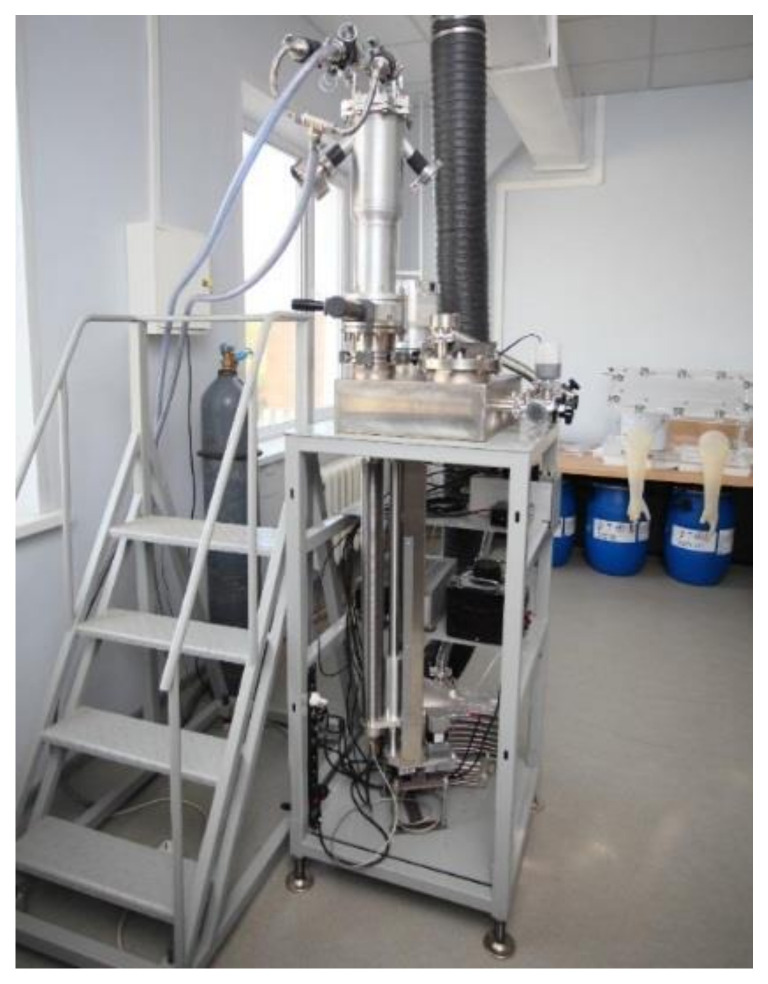
Photo of the lithium deposition system.

The lithium layer is deposited on the target in a separate system by vacuum thermal deposition. A photo of the system is shown in Figure 2. Lithium is stored in sealed test tubes in an argon atmosphere inside an MB-200MOD glove box (MBRAUN, Germany). Before deposition, lithium is taken out of the test tube, and the required amount is weighed, then it is taken out of the glove box through the airlock and placed on a heating plate of the deposition system. The target unit is mounted on the system, and the gate is opened. The vacuum pumping begins. Then, the heating plate is moved to the target using a vacuum linear motion feedthrough, and the heater is turned on. Heating leads to lithium evaporation and its deposition on the copper substrate of the target, cooled by compressed air. After complete evaporation of lithium, the heater is turned off, the plate is pushed back and returned to its original position, and the gate is closed. Then the target unit is removed from the deposition system and, while maintaining the vacuum inside, is transferred to the accelerating neutron source and connected to it.

### 2.2. Efficiency Calibration of Gramma-Ray Spectrometer

The initial calibration of the sensitivity of the HPGe-spectrometer was carried out by two γ-radiation sources placed instead of the target. The first is with radionuclide cesium-137 of the IGI-Ts-3-1 type (Mayak, Russia) with an activity of 4.52 × 10^6^ Bq, and the second is GBa3.11 based on the radionuclide barium-133 (Riverc, Russia) with an activity of 6.86 × 10^5^ Bq. Since the energies of the γ-emission line of radiation sources differ from 478 keV (661 keV for the first source and 383 keV for the second) and the confidence limits of the total error in determining the activity of sources are significant (10% for the first, and 20% for the second), the detector sensitivity was determined with an error worse than 20%. This accuracy is clearly insufficient to evaluate the target.

An increase in the accuracy of the detector sensitivity calibration along the 478 keV photon emission line up to 4% was achieved using seven reference radionuclide photon sources from the OSGI-RT set (Riverc, Russia): ^137^Cs, ^60^Co, ^133^Ba, ^54^Mn, ^152^Eu, ^207^Bi, and ^22^Na. For each of them, the confidence limits of the total error in determining the activity are 3%. These photon sources have a size of less than 1 mm in diameter and can be considered as point sources. Since the activity of these sources is lower by an order of magnitude (from 3.3 × 10^4^ to 1.01 × 10^5^), the sources are placed closer to the spectrometer. They are fixed on a Plexiglas plate mounted on a movable controlled 2D table, moved coaxially close to the entrance window of the detector, and measurements are conducted. The measurements are repeated with the source moved away from the detector up to a distance of 112 mm with a step of 8 mm. The measurements were carried out using 23 γ-radiation lines.

**Figure 3:**
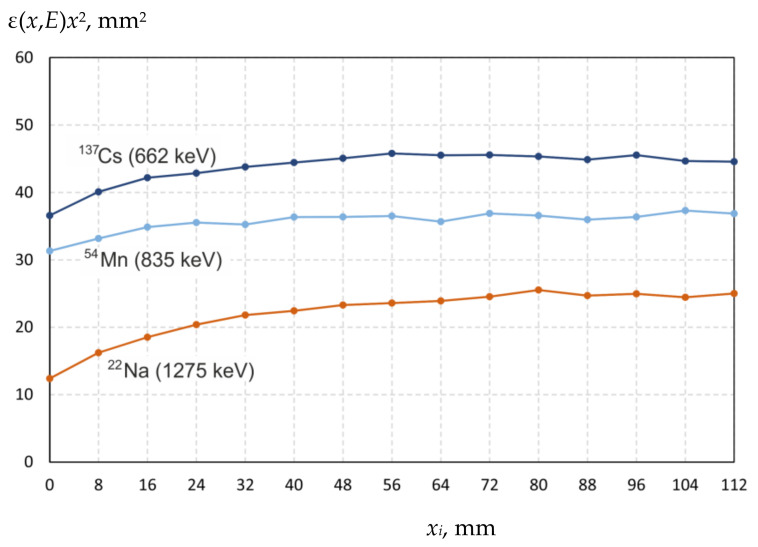
The dependence ε(*x*,*E*)*x*^2^ for ^137^Cs, ^54^Mn, and ^22^Na sources of photon radiation.

Let us assume that the detection efficiency ε(*x*,*E*) is inversely proportional to the square distance from the radiation source to the germanium crystal of the detector *x* = *x*_0_ + *x_i_*, where *x*_0_ is the distance from the surface of the input window to the germanium crystal, *x_i_* is the distance from the surface of the input window to the radiation source (from 0 to 112 mm with 8 mm step), and *E* is the photon energy. Figure 3 shows a graph of the ε(*x*,*E*)*x*^2^ dependence for three sources photon radiation at *x*_0_ = 33 mm.

At large distances, the function ε(*x*,*E*)*x*^2^ is constant (Figure 3), but as the radiation source is moved closer to the detector, the values of the function ε(*x*,*E*)*x*^2^ decrease. This behavior at close distances can be explained by several reasons: significant pulse pile-up at very high count rates, edge effects of the detector and others. Thus, in the measurement of the ^54^Mn source radiation intensity, when the detector dead time does not exceed 11%, the ε(*x*,*E*)*x*^2^ function is almost constant, starting from a distance of 16 mm. In the measurement of the intensity of ^22^Na source with higher activity, when the dead time exceeds 40%, the ε(*x*,*E*)*x*^2^ function becomes constant only from the distance of 56 mm. Consequently, the detector software does not provide reliable measurement of the radiation intensity at high loadings, and measurements carried out with a dead time of more than 5% are unreliable. Note that the dead time correction has been applied by software of HPGe-spectrometer automatically.

We assume that the measurements carried out at a distance *x_i_* ≥ 56 mm are reliable and let us determine the mean values and standard deviations of the ε(*x*,*E*)*x*^2^ function for all 23 γ-radiation lines. By varying *x*_0_, we found the minimum standard deviation sum of the function ε(*x*,*E*)*x*^2^—as *x*_0_ = 33.3 mm. Further, this value is used to determine the efficiency of the registration.

**Figure 4:**
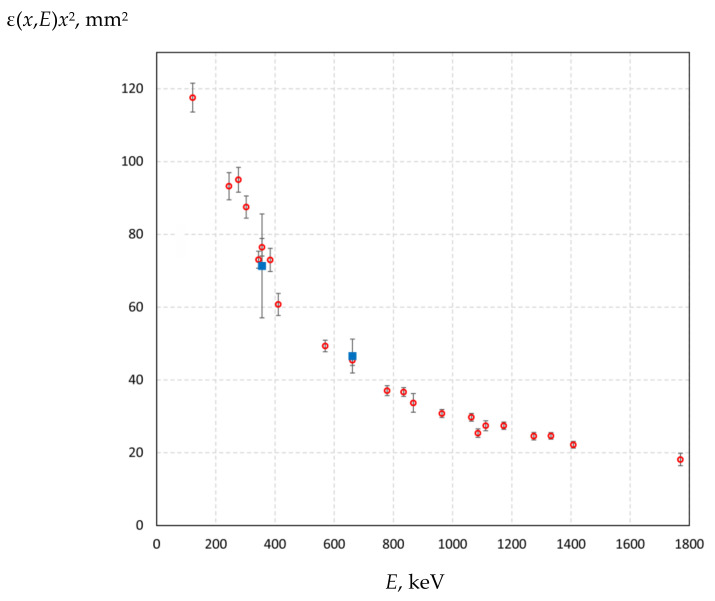
The dependence of the function ε(*x*,*E*)*x*^2^ on the photon energy *E*: ○—standard radionuclide sources of photon radiation from the OSGI-RT set, ■—γ-radiation sources with barium-133 and cesium-137 radionuclides.

The result of measuring the function ε(*x*,*E*)*x*^2^ for 23 γ-radiation lines is shown in Figure 4. The accuracy of the ε*x*^2^ values, which takes into account the passport confidence limit of the error in determining the activity, ranges from 3.2% to 5% with two exceptions (7.5% and 9% at energies of 867 keV and 1770 keV). Finally, the value of the ε(*x*,*E*)*x*^2^ function for the 478 keV γ-radiation line is determined to be 55.5 ± 2 mm^2^.

### 2.3. Calibration of the Proton Beam Energy and Current

The proton energy is determined as *E* = *e*·*U*_H−_ + 2·*e·U*_hv_, where *e* is the electron charge; *e U*_H−_ is the energy of negative hydrogen ions injected into the accelerator (usually 22 keV); *U*_hv_ is the potential of the high-voltage power supply. The potential is measured with a resistive divider installed inside the high-pressure tank of the high-voltage power supply.

Since the ^7^Li(p,n)^7^Be reaction has the threshold energy of 1882 keV, the neutron yield strongly depends on the proton energy. Hence, with a 1% decrease in the proton energy of 2 MeV, the neutron yield decreases by 18% and by 11% at 2.2 MeV.

The potential is measured at a frequency of 1 Hz and is used in the control program of the facility to maintain the potential of the high-voltage electrode of the accelerator at a predetermined level. During the accelerator operation, it was previously noticed that the potential of the high-voltage power source and, consequently, the proton energy gradually decrease during the operational hours. This may be caused by a disproportionate change in the resistances of the resistive divider legs when it is heated. Thus, studying the radiation blistering effect on the neutron yield, it was found that during 5 h of continuous operation of the accelerator at a current of 0.5 mA, the proton energy decreased by 0.65%.

The resistive divider is calibrated according to the ^7^Li(p,n)^7^Be threshold reaction. The BDMN-100-07 detecting unit (Doza LLC, Moscow, Russia) measures the dependence of the neutron radiation dose rate on the proton energy in the range from 1.910 MeV to 2.000 MeV. In this energy range, the dependence of the neutron yield on the energy is linear [5]; the reliability of the linear approximation of values, calculated with a step of 0.01 MeV, *R*^2^ = 1. The value of *R*^2^ was obtained using the standard Excel Pearson function. Note that this straight line of the linear approximation of the data crosses the *x*-axis at 1.885 MeV rather than at the reaction threshold value of 1.882 MeV.

The calibration used in these studies was carried out earlier. Since the preliminary estimates of the measured neutron yield were not consistent with the calculated ones, the calibration of the resistive divider was repeated after the studies. It was found that the measured proton energy was 30 ± 10 keV higher than the true value in the 2 MeV area.

**Figure 5:**
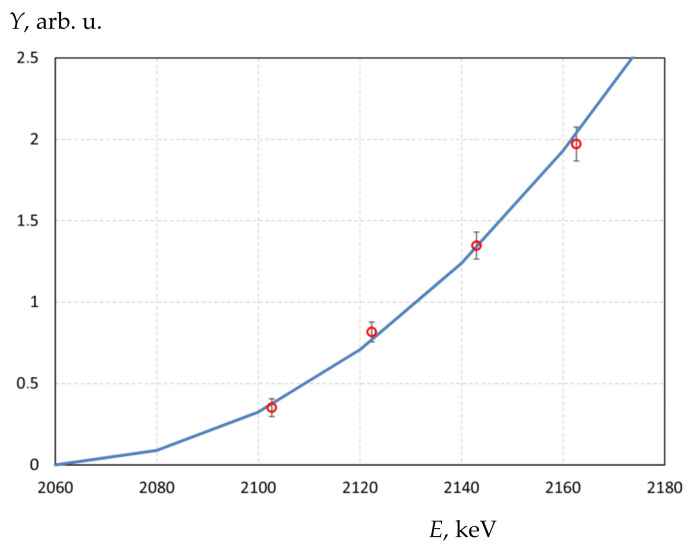
The dependence of the neutron yield *Y* on the proton energy *E* in the ^9^Be(p,n)^9^B reaction: ○—measured, solid line—calculated by the PINO program [6].

In the 2.2 MeV region, the proton energy was additionally calibrated using the ^9^Be(p,n)^9^B reaction (the reaction threshold is 2.057 MeV). To this end, a beryllium disk with a diameter of 32 mm and a thickness of 9.7 mm was tightly attached to the copper substrate of the target through an indium-gallium alloy and then irradiated with a proton beam in position *A* (see Figure 1). A neutron detector with a lithium-containing GS20 scintillator (The Saint-Gobain Crystals, Hiram, OH, USA) was installed in front of the target, the dependence of the recorded signal on the proton energy was measured. The measurement results and the dependence of the neutron yield on the energy calculated by the PINO program [6] are shown in Figure 5. In this figure, the *x*-axis shows the energy *E* obtained by multiplying it by the coefficient *k* selected so that the experimental data fit the calculated ones in the best way, rather than the energy *E* given by the control program of the apparatus. In this case, *k* = 0.983 ± 0.001. This means that if the control program of the apparatus sets the proton energy as 2200 keV, the proton energy was, in fact, 2163 ± 2 keV. If the program sets the proton energy as 2000 keV, the proton energy was, in fact, 1966 ± 2 keV.

Thus, we have calibrated the resistive divider, and in further calculations, a correction will be made for the proper energy value. It should be noted that this correction is applicable only for the mode when the apparatus has worked for some time and the high voltage source is heated. This is not so at the onset of the process. In order to clarify it, the following experiment was carried out.

The irradiation of targets Nos. 12 and 13 (see Table 1) was performed with the actual constant proton energy rather than the set one. To this end, a bending magnet was used as an energy analyzer. If the actual proton energy decreases, the proton beam on the surface of the lithium target shifts. By increasing the set energy in the control system of the apparatus, the proton beam was returned to its original position. The position of the proton beam in the center of the target was controlled by the following diagnostic tools: (i) a video camera recording lithium luminescence under the action of protons; (ii) five thermocouples located inside the copper disk of a lithium target (one in the center, the other four at a distance of 18 mm from the center, uniformly distributed in azimuth); and (iii) according to the readings of two small neutron detectors with a polystyrene-based scintillator enriched with boron, which were glued to the vacuum chamber of the target assembly near the lithium target. To improve the sensitivity of diagnostic tools to proton beam displacement, the proton beam scanner installed in the vertical beam transport path was not turned on. In this case, the proton beam on the target surface had a characteristic size of 3 cm.

**Figure 6:**
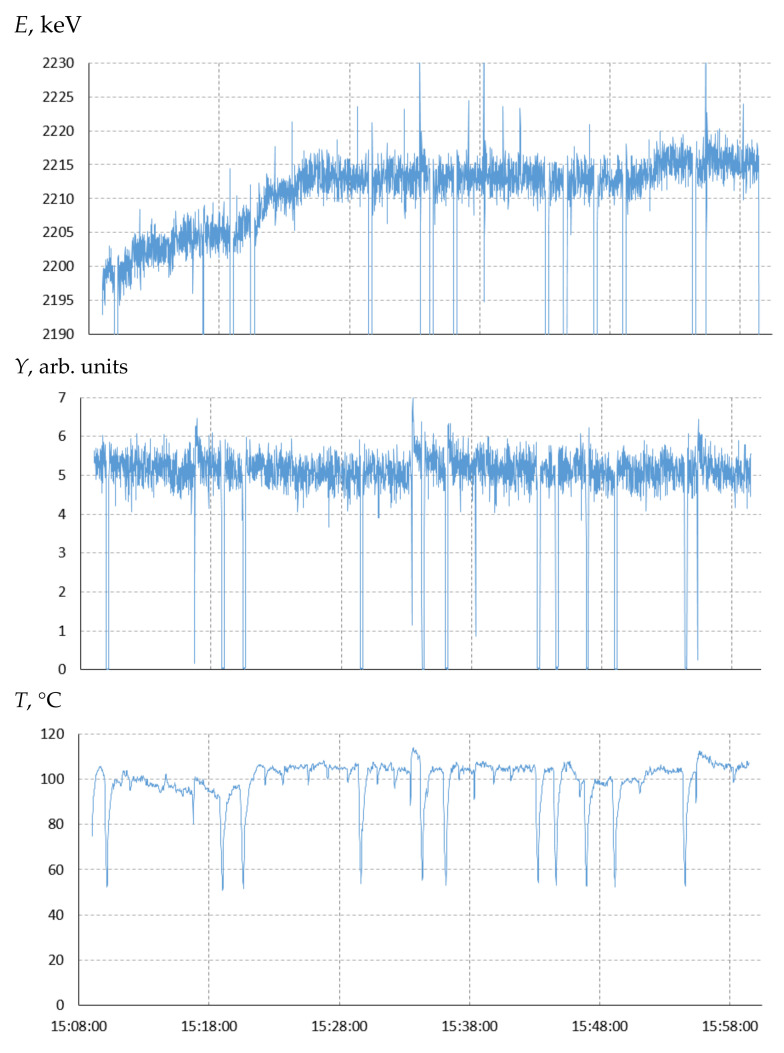
The time dependence of the proton energy *E*, the neutron detector signal *Y*, and the temperature in the center of the lithium target *T* during the irradiation session of the target No. 12.

Figure 6 shows the time dependence of the proton energy, the neutron detector signal, and the temperature in the center of the lithium target during the irradiation session of target No. 12. It can be seen that the neutron yield and temperature in the center of the target were practically constant, while the set proton beam energy had to be practically linearly increased during the first 15 min of irradiation at 13 keV. In 15 min, no further energy correction is required to maintain the proton beam at the center of the target. The exposed proton energy in the plateau mode was 2213 ± 2 keV. By multiplying it with the coefficient *k* = 0.983 ± 0.001, we obtain the actual proton energy during the irradiation of target No. 12; it is 2175 ± 3 keV. Let us immediately pay attention to the fact that at the very beginning of irradiation, the actual proton energy was equal to 2175 keV since the proton beam hit the center of the target. This means that at the very beginning of irradiation, the coefficient *k* = 2175/2200 = 0.9886.

It should be noted that the accelerator operating mode at 2.2 MeV was practically limiting and was accompanied by 11 high voltage breakdowns (see temperature data in Figure 6), after which the proton beam parameters returned to their initial values in 15 s.

A similar procedure for determining the correct proton energy was applied in the irradiation session of target No. 13.

For the other irradiation sessions, when the constancy of the proton energy is not maintained by the bending magnet used as the energy analyzer, it is necessary to make yet another assumption: the coefficient *k* depends on time. In the first 15 min, it decreases linearly from 0.9886 (the estimate is given above) to 0.983 and then remains constant for 45 min. Thus, the weighted average coefficient *k* of irradiation sessions for targets Nos. 1–11 is 0.98375 ± 0.002. We will use it to correct the proton energy in the irradiation sessions of these targets.

The proton current is measured by the voltage drop across a calibrated resistance connected to a target unit electrically isolated from the apparatus. Although the target unit is made in the form of a deep Faraday cup, emission of secondary electrons from it is possible. To measure electron emission, the gate as a part of the target unit was additionally isolated, and potential was applied to it. The application of a 500 V negative blocking voltage reduces the proton current by an average of 1%, which means that the measured proton fluence is overestimated by 1%.

## 3. Results and Discussion

To determine the neutron yield, we measured the equivalent production of radioactive isotope ^7^Be in the ^7^Li(n,p)^7^Be reaction. This requires the calculation of 4π radioactivity of ^7^Be from the activity measured at a very small solid angle with an efficiency calibrated γ-ray detector. As a result of electron capture, the radioactive ^7^Be atomic nucleus is converted back to ^7^Li with a half-life of 53.22 days. In 10.3% of cases, the decay is accompanied by the emission of a 478 keV photon. If we prevent beryllium propagation from the lithium target, then the measurement of the target activation makes it possible to determine the number of produced ^7^Be nuclei, which is equal to the number of generated neutrons.

### 3.1. Measurement of Target Neutron Yields

In the research, 13 targets were used. Firstly, the target is irradiated with a proton beam, usually for 1 h at a current of 1 mA. The next day, the target activation is measured with an HPGe-spectrometer. To this end, the target unit is disassembled: the lithium target is removed from it, put in a transparent bag, and placed at a distance of 1854 mm from the entrance window of the spectrometer, then its activity is measured. In this case, irradiated targets can be considered as point sources. For each target, the measurement lasts for approximately 7 min (this time was not taken into account in calculations). The activity of the target unit is also measured to determine the amount of beryllium transferred from the target. The fraction of beryllium transferred from the target did not exceed 1/10,000.

A total of 15 sessions of neutron generation were carried out; 13 lithium targets were used. Only new copper substrates were used, and a new lithium layer was always deposited while making all 13 targets. Basic data on the irradiation sessions are presented in Table 1. The results of measuring the number of ^7^Be atomic nuclei are presented in Table 2.

Let us describe the procedure of getting the results. Starting from the common formula for the activity of a radioactive source, which describes the number of decays per unit of time, and taking into account the detector efficiency at 1854 mm distance—ε, one obtains: $A=A_{m}\cdot \exp\left(\frac{T_{\frac{1}{2}}}{0.693\cdot \Delta t}\right)\cdot \frac{1}{\varepsilon },$ where *A*—activity at the end of irradiation; *A_m_*—the measured activity; *T*_1/2_—half life; Δ*t*—the delay between end of irradiation and start of measuring. Knowing the activity of the radiation source, one determines the total number of radioactive nuclei *N* and, hence, neutrons produced from the following ratio: $N=\frac{T_{1/2}}{0.693}\cdot \frac{A}{0.103},$ taking into account that the decay of the atomic nucleus of beryllium is accompanied by the emission of a 478 keV photon in only 10.3% of cases.

On the example of target No. 4, we show how the result was obtained. The count rate of the 478 keV line *A_m_* was 63.13 s^−1^, the measurement accuracy was 0.6%, and the dead time was 1.4%. Since the absolute sensitivity of the HPGe-spectrometer along the 478 keV line is 1.56 ± 0.07 × 10^−5^ when the radiation source is located at a distance of 1854 mm and dividing the count rate by the sensitivity, we obtain the radiation intensity of 478 keV photons: 63.13/1.56 × 10^−5^ = 4.07 × 10^6^ s^−1^. Since the decay of the beryllium atomic nucleus is accompanied by emission of a 478 keV photon only in 10.3% of cases, and dividing the obtained radiation intensity of 478 keV photons by 0.103, we obtain the target activity value *A* = 4.07 × 10^6^/0.103 = 3.93 × 10^7^ Bq. Knowing the radiation source activity, we can determine the number of radioactive nuclei *N* from the following ratio: *N* = *A T*_1/2_/ln2, where *T*_1/2_ = 53.22 days = 4.6 × 10^6^ s, ln2 = 0.693. Therefore, *N* = 3.93 × 10^7^ × 4.6 × 10^6^/0.693 = 2.606 × 10^14^. Since the measurements were carried out later rather than immediately after the end of neutron generation (in 1 day and 1 h), some of the beryllium nuclei decayed. According to the law of radioactive decay, the number of non-decayed atoms at the time t is related to the initial (at the time *t* = 0) number of atoms by the ratio $\frac{N\left(t\right)}{N_{0}}=e^{-\frac{0.693\;t}{t_{1/2}}}$. At *t* = 9 × 10^4^ s (25 h), *N*(*t*) = 0.9865 *N*_0_. Consequently, at the end of neutron generation, the lithium target contained 1.35% more atomic nuclei of beryllium-7 than at the time of the measurement (2.642 × 10^14^). Since neutron generation lasted for 1 h, part of the beryllium-7 nuclei decayed at the time of generation. Assuming the current to be constant, we find that 0.027% of the formed beryllium-7 nuclei decayed by the end of generation. Taking into account this small correction, we obtain the total number of nuclei formed in lithium target No. 4 as 2.642 × 10^14^.

Assuming the half-life and the probability of 478 keV photon emission to be accurate tabular values, the error in measuring the number of generated neutrons is determined by the absolute sensitivity of the detector and the accuracy of measuring the emission line intensity—it is 5%.

Since the proton energy is found knowing the proton fluence, let us determine the neutron yield using the PINO program [6]. When calculating the neutron yield, let us take into account the lithium-7 percentage. For making the targets, two types of lithium are used: natural lithium and lithium enriched with ^7^Li isotope produced by Novosibirsk Chemical Concentrates Plant (Russia). In the fraction of natural lithium, the lithium content was 99.956%; the remaining 0.044% are impurities of Na, K, Ca, Mg, Mn, Fe, Al, SiO_2_, and N. The content of isotope 7 in natural lithium varies from 92.41% [7] to 92.58% [8]. We further assume the lithium-7 content in natural lithium to be equal to the average value of 92.5%. In enriched lithium, the lithium-7 atomic fraction was 99.988%; the rest is practically the same impurities.

### 3.2. Influence of Impurities in the Li-Targets

The natural lithium used contains impurities at a concentration of 0.044%. The weighted average value of the atomic number of impurities is 15, according to the product data sheet. This value is 5 times higher than the atomic number of lithium, and therefore, proton deceleration determined by electron interaction is 5 times more effective on impurities than those on lithium. This means that impurities in a concentration of 0.044% reduce the neutron yield by 0.22%. In lithium enriched with isotope lithium-7, the concentration of impurities is 0.012%, which means that their presence reduces the neutron yield by 0.06%. All these estimates are correct if we suggest that when lithium is deposited on the target, the impurities are also deposited.

To check the assumption, the following experiment was carried out. An alpha-spectrometer based on a PDPA-1K silicon detector and a TsSU-1K digital spectrometric device (IPTP, Russia) is installed on one of the branch pipes of the target unit (see Figure 1). The spectrometer was calibrated with a reference OSAI source based on radium-226 radionuclide. The spectrometer was used to measure the energy spectrum of protons scattered at an angle of 168°.

**Figure 7:**
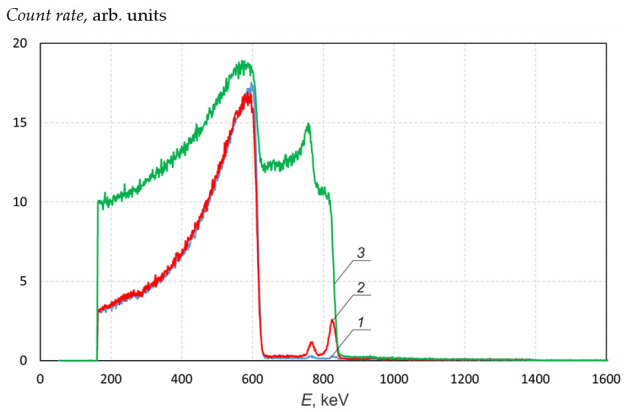
Signals of the alpha-spectrometer upon 1 MeV proton irradiation of the freshly deposited lithium layer on the target (*1*) and the lithium layer after deliberate admission of atmospheric air into the target unit for 10 min (*2*) and for 1 h (*3*).

Figure 7 shows the signal of the alpha-spectrometer irradiated with 1 MeV protons from the freshly deposited lithium layer of the target. It can be seen that proton reflection occurs from lithium (proton energy below 630 keV), carbon (peak around 768 keV) and oxygen (825 keV); there are no heavier impurities; a small plateau up to 1400 keV is caused by alpha particles of the ^6^Li(p,α)^3^He reaction. To calculate the content of impurities, the SIMNRA v.7.03 program (Max Planck Institute for Plasma Physics, Germany) [9] was used. The SIMNRA database allows the simulation of non-Rutherford backscattering, nuclear reaction analysis, and elastic recoil detection analysis. Using SIMNRA, we determined that on the surface of the lithium layer, there was lithium oxide with a density of 2.4 × 10^16^ atoms/cm^2^ (~6 nm of lithium oxide with the crystal density) and carbon over it with the density of 6 × 10^15^ atoms/cm^2^ (~0.5 nm of carbon with the crystal density). The appearance of these layers is most likely due to the interaction of lithium with the residual gas. To confirm this assumption, atmospheric air was admitted into the target unit, first for 10 min and then for another 50 min so that the exposure time was 1 h. It can be seen that exposure to air for 10 min leads to an increase in carbon and oxygen concentration on the surface of the lithium layer, and with a longer exposure of the target to air, oxygen penetrates into the deep lithium layers.

After admitting atmospheric air into the target unit for 10 min, the neutron yield measured by a neutron detector with a GS20 lithium-containing scintillator (The Saint-Gobain Crystals, USA) and BDMN-100–07 detectors (Doza, Russia) decreased by 5%. Since the signal of protons scattered from carbon and oxygen is 130 times greater, in this case, the presence of oxygen and carbon on the freshly deposited lithium layer should lead to a 130-times decrease in the neutron yield, namely by 0.03%.

Thus, it was found that during the deposition of a lithium layer, impurities contained in lithium did not deposit; however, carbon and oxygen were adsorbed on the lithium surface. Carbon and oxygen adsorption leads to a decrease in the neutron yield by 0.03% and can be reduced by improving the vacuum conditions in the target unit.

### 3.3. Summary of Results

As mentioned above, if we prevent beryllium propagation from a lithium target, then the measurement of the activation of the target allows us to determine the number of ^7^Be nuclei produced during neutron generation. The number of ^7^Be nuclei is equal to the number of neutrons generated. In this study, not only the activation of the lithium target but also the activation of the target unit from which the target was removed were measured. It was found that a part of beryllium-7 was transferred from the target to the target unit, but this part is small—less than 1/10,000 in all irradiation sessions. Since the transfer of radioactive isotope beryllium-7 from the target is small, the proposed method for measuring the neutron yield is applicable.

**Figure 8:**
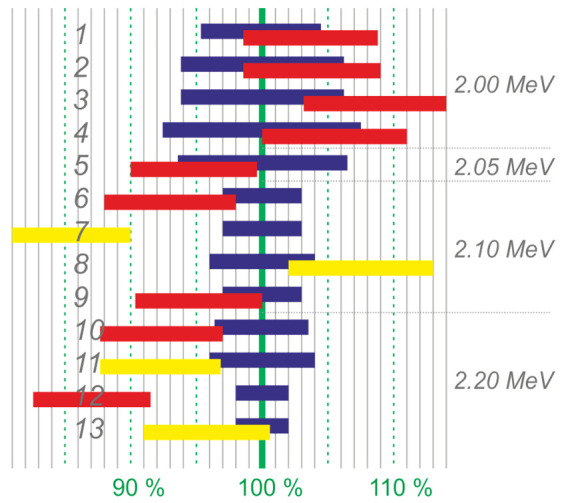
A comparison of the measured neutron yield with the calculated one. From top to bottom—targets in order, blue rectangles—intervals of the calculated neutron number, red and yellow—intervals of the measured neutron number (red—natural lithium, yellow—enriched with lithium-7 isotope). On the right: the characteristic values of the proton energy.

Taking into account all the corrections, we present the final result of the study in Table 3. This table shows the measured number of beryllium-7 nuclei formed in the lithium layer of the target as a result of the ^7^Li(p, n)^7^Be reaction for each target, the correct value of the proton energy that irradiated the lithium target, the calculated number of generated neutrons, and the ratio of the measured number of beryllium-7 nuclei to the calculated number of neutrons in percentage. The result is graphically shown in Figure 8.

It can be seen that in 11 out of 13 irradiation sessions, the measured value of the neutron yield agrees with the calculated one. In two irradiation sessions, the measured neutron yield did not coincide with the calculated one. If in the irradiation session of target No. 7, the reason is clear (during irradiation some holes formed in the lithium layer), then in the irradiation session of target No. 12, the reason remains unclear. Note that the problem of hole formation during the first irradiation of the lithium-7 target was promptly solved by correcting the lithium deposition procedure, and no such phenomenon was observed later.

It can be seen that the measured amount of beryllium-7 is always slightly higher than the calculated one if the target is placed in the horizontal proton beam transport tract (Figure 1 *A*) (sessions 1–3 and 8) and is usually less if the target is placed in the vertical tract (Figure 1 *B*) (sessions 4–7 and 9–12). Perhaps, this is caused by the large size of the proton beam and its displacement on the target surface, due to which part of the proton beam could fall outside the lithium deposition area, although this was not seen visually in the beam imprint on the lithium surface.

## 4. Conclusions

Currently, several clinics with the accelerator-based neutron sources and lithium targets are putting BNCT into operation to treat patients with malignant tumors. For planning the therapy, it is important to confirm the neutron yield in the ^7^Li(p,n)^7^Be reaction. In this work, the neutron yield from a lithium target was measured by its activation with radioactive isotope beryllium-7 using a γ-radiation spectrometer based on a semiconductor detector made of high purity germanium. The agreement of the measured yield with the calculated one is shown, which is important for planning the treatment.

## Figures and Tables

**Table 1:** Data on irradiation sessions. Columns: 1—serial number of targets; 2—neutron generation duration *t*; —type of lithium used (natural or enriched with lithium-7 isotope); 4—target position *A* or *B* (see Figure 1); 5—proton energy *E*; 6—average current *I*; 7—approximate size of the proton beam on the surface of the lithium target *d*; 8—collected proton fluence *F* measured with the Faraday cup.

1	2	3	4	5	6	7	8
No.	*t*	Lithium	Position	*E*, keV	*I*_avr_, µA	*d*, cm	*F*, C
1	59 min 56 min	^nat^Li	A	1973 ± 4	1033 ± 16 1070 ± 30	1.7	7.20
2	1 h 2 min 57 min	^nat^Li	A	1973 ± 5	1056 ± 30 1050 ± 23	1.7	7.20
3	56 min	^nat^Li	A	1973 ± 5	1060 ± 20	1.7	3.60
4	1 h	^nat^Li	B	1967 ± 6	1006 ± 27	4	3.53
5	59 min	^nat^Li	B	2020 ± 8	1018 ± 34	4	3.60
6	56 min	^nat^Li	B	2065 ± 5	1027 ± 60	4	3.60
7	1 h	^7^Li	B	2069 ± 6	1017 ± 40	4	3.60
8	21 min	^7^Li	A	2067 ± 5	1024 ± 11	1.7	1.00
9	1 h 4 min	^nat^Li	B	2069 ± 6	2021 ± 90	4	7.20
10	49 min	^nat^Li	B	2162 ± 6	1295 ± 140	4	3.60
11	1 h 8 min	^7^Li	B	2172 ± 7	1003 ± 48	4	3.60
12	51 min	^nat^Li	B	2174 ± 3	1238 ± 80	3	3.60
13	1 h 3 min	^7^Li	B	2176 ± 3	1010 ± 30	3	3.60

**Table 2:** Data on the measurements of the target. Columns: 1—serial number of targets; 2—time between the end of neutron generation and the beginning of the measurement of the target activity *T*; 3—count rate, s^−1^; 4—number of the formed ^7^Be nuclei, pcs.

1	2	3	4
No.	*T*	Count Rate, s^−1^	^7^Be Nuclei, pcs
1	10 d 23 min; 8 d 2 h 17 min	124.1	5.69 × 10^14^
2	7 d 51 min; 6 d 25 min	127.84	5.70 × 10^14^
3	3 d 1 h 17 min	69.52	2.98 × 10^14^
4	1 d 1 h	63.13	2.64 × 10^14^
5	19 h 1 min	96.48	4.02 × 10^14^
6	20 h 1 min	128.83	5.37 × 10^14^
7	1 d 1 h 22 min	130.16	5.44 × 10^14^
8	2 d 1 h 41 min	44.69	1.89 × 10^14^
9	3 d 1 h 31 min	259.86	11.2 × 10^14^
10	17 h 38 min	217.89	9.07 × 10^14^
11	19 h	250.64	10.4 × 10^14^
12	18 h 31 min	220.5	9.19 × 10^14^
13	18 h 33 min	265.29	11.1 × 10^14^

**Table 3:** Data on the measurements of the target. Columns: 1—serial number of targets; 2—the type of lithium used (natural or enriched with the lithium-7 isotope); 3—measured number of ^7^Be nuclei; 4—the actual proton energy; 5—calculated number of neutrons; 6—the ratio of the measured number of beryllium-7 nuclei to the calculated number of neutrons, %.

1	2	3	4	5	6
No	Lithium	*N*_7Be_, ×10^6^ n/µC Measured	*E*, keV	*Y*_n_, ×10^6^ n/µC Calculated	N_7Be_/*Y*_n_, %
1	^nat^Li	79.0 ± 3.9	1973 ± 4	76.1 ± 3.5	104 ± 10
2	^nat^Li	79.2 ± 4.0	1973 ±5	76.3 ± 4.7	104 ± 11
3	^nat^Li	82.8 ± 4.2	1973 ± 5	76.1 ± 4.7	109 ± 11
4	^nat^Li	74.8 ± 3.7	1967 ± 6	70.8 ± 5.4	106 ± 13
5	^nat^Li	111.7 ± 5.6	2020 ± 8	118.1 ± 7.5	95 ± 11
6	^nat^Li	149.2 ± 7.5	2065 ± 5	159.7 ± 4.7	93 ± 8
7	^7^Li	151.1 ± 7.5	2069 ± 6	176.9 ± 6.1	85 ± 9
8	^7^Li	189 ± 9.0	2067 ± 5	175 ± 5.0	108 ± 8
9	^nat^Li	155.6 ± 7.8	2069 ± 6	163.3 ± 5.8	95 ± 9
10	^nat^Li	251.9 ± 12.5	2162 ± 6	272.8 ± 9.2	92 ± 8
11	^7^Li	288.9 ± 14.4	2172 ± 7	313.1 ± 12.2	92 ± 9
12	^nat^Li	255.3 ± 12.8	2174 ± 3	293.6 ± 4.7	87 ± 7
13	^7^Li	308.3 ± 15.6	2176 ± 3	321.9 ± 5.6	96 ± 7

## Data Availability

The data presented in this study are available on request from the corresponding author.
